# 

**Published:** 1885-01

**Authors:** 


| | PAGE |
|---|---|
| ORIGINAL COMMUNICATIONS.—Introductory Address. Rush Shippen Huidekoper, M.D.,V.S., | 1 |
| Notes on Veterinary Medicine in Great Britain. N. S. Townsend, M.D. | 19 |
| Scarlet Fever and Measles in Dogs and Cats. John C. Peters, M.D. | 21 |
| Enzoötic Abortion in Cows. F. S. Billings, V.S. | 25 |
| Notes, with Commentations, on the Psychology of the Chimpanzee, (Troglodytes Niger), now in captivity at the Central Park Menagerie, New York. Dr. C. Pitfield Mitchell | 38 |
| Gangrene. Joseph M. Skelly, V.S. | 53 |
| The Life Work of Pasteur | 62 |
| A Consideration of the Anthrax Inoculations of Pasteur. Prof. H. Putz | 73 |
| EDITORIAL.—Veterinary Questions in the United States—Remuneration for the loss of Animals Slaughtered on Account of Contagious Diseases—The Facts as to the Stamping Out of Contagious Animal Diseases—Relation of Animal Diseases to the Public Health—Absence of Dr. Billings—Bureau of Animal Industry—The Superior Royal School of Veterinary Medicine of Milan—Veterinary Department, University of Pennsylvania—Koch and his Methods | 86 |
| CASE DEPARTMENT.—Autopsies at Central Park Menagerie—Removal of a Portion of the Teat of a Cow—Cases of Tetanus Successfully Treated—Angina Paralytica in the Horse caused by the Use of Ensilage | 101 |
| PROCEEDINGS OF VETERINARY MEDICAL SOCIETIES.—Massachusetts Veterinary Medical Association—Montreal Veterinary Medical Association | 104 |
| CORRESPONDENCE | 107 |
| REVIEWS | 108 |

